# Characterization of *sck1*, a Novel *Castanea mollissima* Mutant with the Extreme Short Catkins and Decreased Gibberellin

**DOI:** 10.1371/journal.pone.0043181

**Published:** 2012-08-15

**Authors:** Xian-Ping Guo, Xing-Liang Li, Xu-Wei Duan, Yuan-Yue Shen, Yu Xing, Qing-Qin Cao, Ling Qin

**Affiliations:** 1 Key Laboratory of New Technology in Agriculture Application of Beijing, College of Plant Science and Technology, Beijing University of Agriculture, Beijing, China; 2 College of Biotechnology, Beijing University of Agriculture, Beijing, China; 3 College of Forestry and Horticulture, Xinjiang Agricultural University, Urumqi, China; National Taiwan University, Taiwan

## Abstract

A novel Chinese chestnut (*Castanea mollissima* Bl.) mutant with extreme short catkins, here was named *sck1* and has been characterized in the present study. This *sck1* caused 6-fold shorter than wild-type catkins. Endogenous gibberellic acids markedly decreased in the mutant, and application of exogenous GA_3_ could partially restore the *sck1* phenotype to the wild-type phenotype. Paclobutrazol (PP_333_), an antagonist of GAs biosynthesis, could significantly inhibit the wild-type catkins growth, and lead to a short catkins phenotype similar to the *sck1*. In addition, compared to the wild-type catkins, the mRNA expression level of *ent*-kaurenoic acid oxidase (*KAO*), a gibberellin biosynthsis key gene, was significantly down-regulated (*P*<0.01) in the *sck1*. Importantly, transient over-expression of a normal *CmKAO* gene in short catkins also could partially restore the wild-type phenotype. Real-time PCR and semi-quantitative analysis showed that the mRNA expression level of *KAO* was significantly up-regulated. In addition, transient RNA interference of *CmKAO* in wild-type catkins led the mRNA expression level of *KAO* decrease significantly and inhibited the wild-type catkins elongation strongly. Taken together, our results suggest that the lower gibberellic acids content that is due to decreased *CmKAO* expression level may contribute to the generation of the extreme short male catkins, *sck1*.

## Introduction

Chinese chestnut (*Castanea mollissima* Bl.) is a very value plant species with an important role in food, economy and ecology. However, more male flowers and less female flowers (in a proportion of 3000∶1) significantly limits yield in Chinese chestnut [1]. Several previous reports show that thinning 90–95% of the male inflorescences by manual or chemical agent significantly can increase nut yield up to 47% and improve the nut quality [2], [3]. Thus, breeding and selecting chestnut trees with relatively more female flowers is a desirable work in improving chestnut yield. Fortunately, a novel natural bud mutant of Chinese chestnut was found and named *sck1* (short catkins), which was associated with a greater number of female flowers and increased yield [4]. But, the generation mechanism of the *sck1* remains unknown to date.

The molecular mechanism for generation of bud mutation in woody perennial plants is not well understood. Present studies mainly focused on the insertion of retrotransposons [5], [6], DNA methylation [7], and gene structure and expression differences [8]. In nature, numerous dwarf mutants are deficient in the biosynthesis or perception of gibberellic acids (GAs). GAs can regulate plant development processes, such as shoot and stem elongation [9], [10]. Severe GAs-deficient mutants display extreme dwarf and male-sterile phenotypes throughout their life cycles. GAs biosynthesis and catabolism pathways have been studied extensively by gas chromatography-mass spectrometry analysis of GAs contents, purification of GAs metabolism enzymes, isolation of GAs-deficient mutants and cloning of the corresponding genes [9], [11]. Recent molecular genetic and biochemical studies helped to identify GAs biosynthesis and catabolism genes in *Arabidopsis* and other species. A number of GAs-deficient dwarf mutants in *Arabidopsis* were isolated, cloned and identified [12]–[17]. In the last decade, genes encoding enzymes in most steps in the GAs metabolic pathways have also been isolated from different species [9], [11]. But whether GAs also functions in bud mutant in fruit tree are seldom reported [18].

Here, we investigate the extreme short catkins phenotype that is may due to lower gibberellic acids content than wild-type (WT) during catkins development. The endogenous GAs levels are normal in vegetative part but lower in reproductive organ. The GAs contents in young shoot and leaves were no significant difference between wild-type and the *sck1*. The differentiation process of male catkins can be divided into six stages, including formation of the primordium of the male inflorescence, the floral cluster, the individual floret, the floral envelope, the stamen, and the anther. During the catkin development period of the *sck1*, the upper parts became processing yellow, curved, and finally dropped off, while only several basal catkins including female catkins still developed normally, and the process of cell death in *sck1* catkins had the typical characteristics of programmed cell death (PCD) [4].

The wild-type male catkins development can be suppressed by the gibberellin antagonist, paclobutrazol (PP_333_) and the catkin length of *sck1* can be partially restored by GA_3_ application treatment. The content of gibberellic acids are markedly lower in *sck1* than wild-type catkins, though other phytohormones are not significant changes between the short catkins mutant and wild-type catkins. The analysis for the expressions of gibberellin biosynthesis related genes show that the gene expression of *KAO* (HQ658173) but not the other genes is distinctly decreased in *sck1* compare to in wild-type. We cloned *CmKAO* cDNA from wild-type catkins. The transient over-expressing *CmKAO* in *sck1* catkins could partially rescue the short catkins phenotype and transient RNA interference of *CmKAO* in the wild-type catkins could inhibit the wild-type catkins strongly. Those results indicated that the lower gibberellic acids content that is due to decreased *CmKAO* expression level may contribute to the generation of the extreme short male catkins, the *sck1*.

## Results

### Identification of Short Catkins Mutant

**Figure 1:**
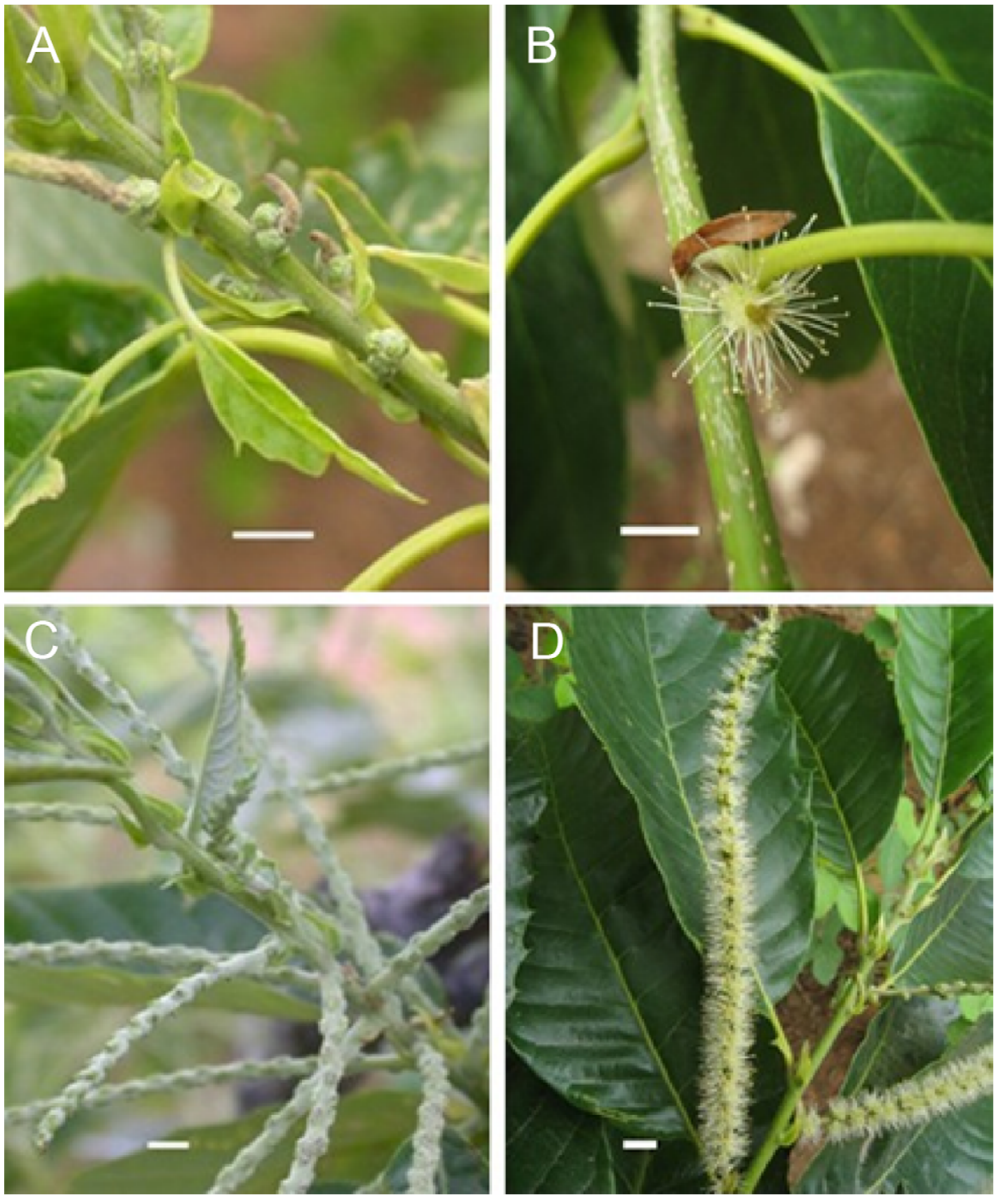
Comparison of catkin length between the mutant catkins and WT. (**A**) mutant catkins before flowering. (**B**) mutant catkin in flowering. (**C**) wild-type catkins before flowering. (**D**) wild-type catkins in flowering. Bar  = 1 cm.

The chestnut mutant of *sck1* was originally found in an orchard in Miyun (Beijing, China) in the 1995 s. It occurred spontaneously from the Chinese chestnut as a bud mutation. The mutant has been propagated by grafting onto different rootstocks, and could remain stable phenotype under field condition. The observation for several years showed that the mutant catkins were significantly shorter than wild-type catkins (Fig. 1).

### Endogenous GAs Level in Wild-type Catkins and *Sck1*

**Figure 2:**
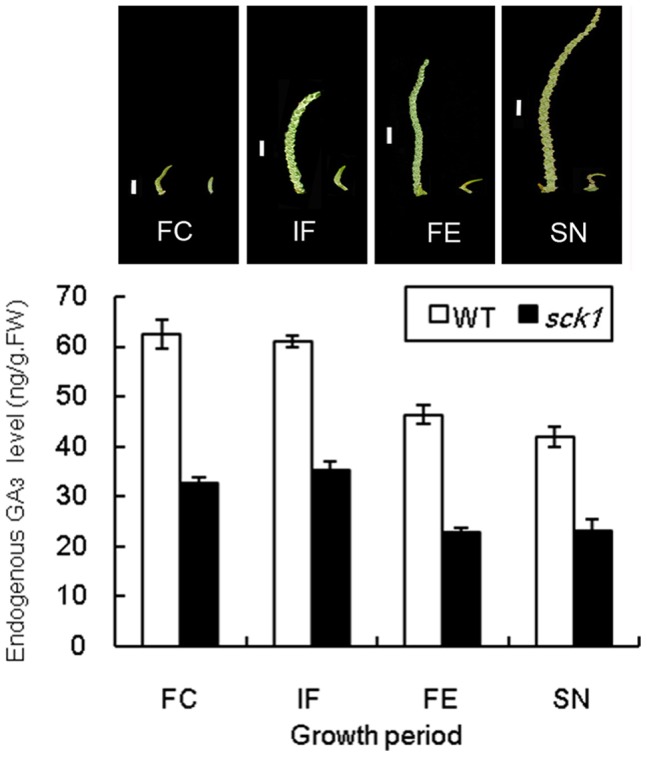
The phenotype of WT and *sck1* and dynamic patterns of GA_3_ content during the catkin development. Formation of the primordium of the floral cluster (FC), the individual floret (IF), the floral envelope (FE) and the stamen (SN). The catkins of *sck1* became yellow in FE and became curved in SN. Catkins were gathered in the four periods, and the endogenous GA_3_ were determined by LC/MS. Data are means (±SE, n = 3).

**Figure 3:**
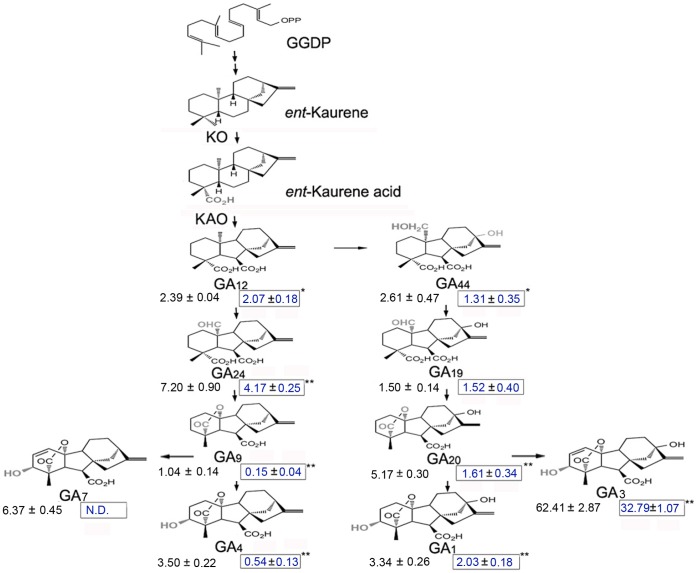
Endogenous GAs level in the WT (left) and *sck1* (right, boxed). Catkins were gathered in the formation of the primordium of the floral cluster, and the endogenous GAs were determined by LC/MS. N.D., not detected due to low abundance; one asterisk indicates significant difference at *P*<0.05 compared with the wild type and two asterisk indicates significant difference at *P*<0.01. Data are means ±SD from three trials (ng/g fresh weight).

In order to explore whether which plant hormones are responsible for this mutant, GA_3_, Ethephon, Abscisic acid (ABA), Indole-3-acetic acid (IAA), Brassinolide and Cytokinin (CTK) were used in feeding experiment [19]. While only GA_3_ could avoid programmed cell death in *sck1* and rescue the phenotype of short catkins partially. This result showed that gibberellic acids play a very important role in the *sck1* catkins development. Endogenous GA_3_ levels from the primordium formation of the floral cluster to the primordium formation of the stamen were determined by liquid chromatography – mass spectrometry (LC/MS) (Fig. 2). The dynamic patterns of GA_3_ levels in WT and *sck1* are very similar, but, obviously, the lower levels of GA_3_ were observed in *sck1*, and has a significant difference (*P*<0.01) in four periods. Besides, some more endogenous GAs (i.e., GA_1_, GA_4_, GA_7_, GA_9_, GA_12_, GA_19_, GA_20_, GA_24_ and GA_44_) in wild-type catkins and *sck1* were determined. As shown in the schematic representation of GA biosynthesis (Fig. 3), all endogenous GAs tested except GA_19_ were significantly lower in *sck1* than in wild-type catkins, which further identified the correlation between GAs and *sck1* development.

**Figure 4:**
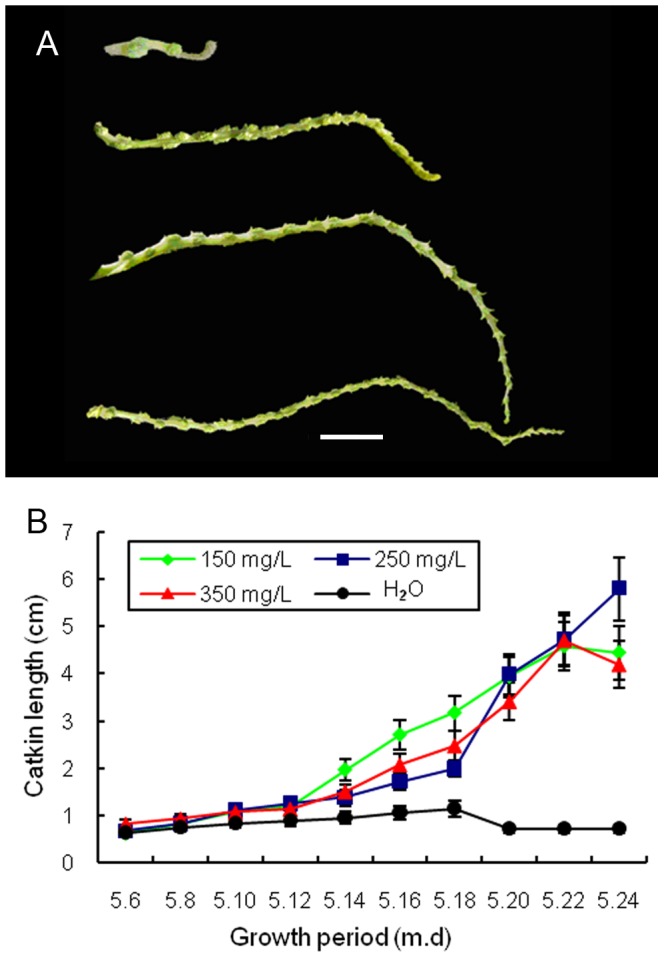
Exogenous GA_3_ treatment on the *sck1* catkins. (**A**) The *sck1* catkins from up to down were treatment by H_2_O, 150 mg/L, 250 mg/L and 350 mg/L of GA_3_ on mutant catkins every two days during growth period in year 2007 and length changes were recorded. Bar  = 1 cm. (**B**) The average length of *sck1* catkins after different concentrations of GA_3_ treatment. Data are means (±SE, n = 30).

In order to further identify the effect of exogenous GAs on resuming growth of short catkins, the *sck1* catkins were sprayed with GA_3_ on different concentrations in year 2007, and the elongation growth were observed (Fig. 4). The significant difference on catkin length was clearly visible in the second week after the first GA_3_ treatment. On average, the short catkins elongated to 5.8 cm on the whole treatment period from May 6 to May 24, while the control elongation is only about 0.73 cm. The most obvious elongation up to 17.6 cm was observed in 250 mg/L of GA_3_, about 24 times of the control. The pollen viability of *sck1* after application of exogenous GA_3_ was not significantly different compare to wild-type (data not shown).

**Figure 5:**
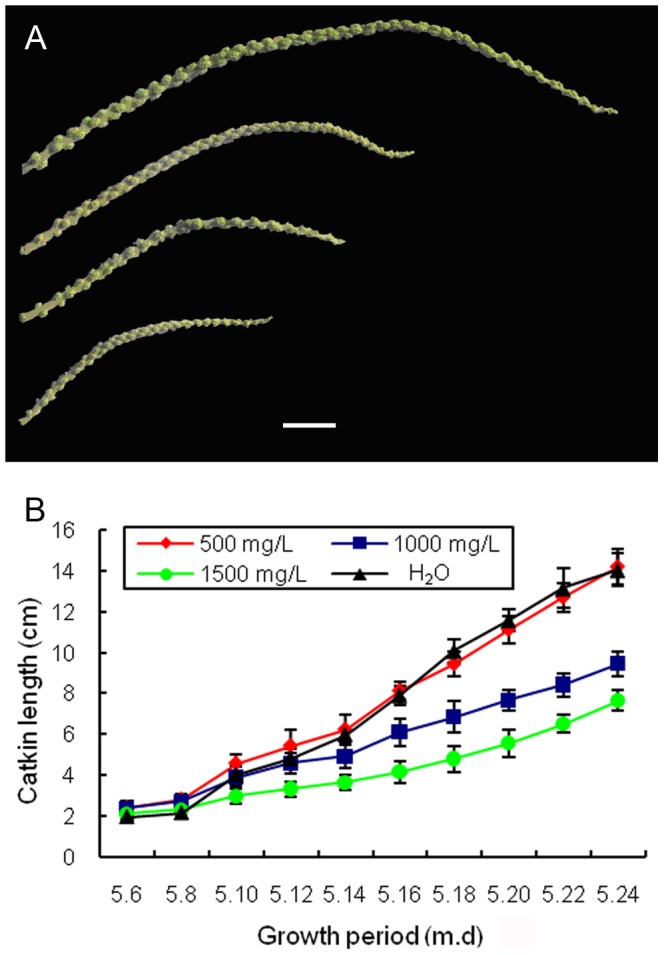
PP_333_ treatment on the wild-type catkins. (**A**) Catkins from up to down were treatment by H_2_O, 500 mg/L, 1000 mg/L and 1500 mg/L of PP_333_ on wild-type catkins every two days during growth period in year 2007 and length changes were recorded. Bar  = 1 cm. (**B**) The average length of wild-type catkins after different concentrations of PP_333_ treatment. Data are means (±SE, n = 30).

In contrast, PP_333,_ a gibberellin antagonist, which inhibit specifically the oxidation of *ent*-kaurene to *ent*-kaurenoic acid [20] was applied on the wild-type catkins in the same period with the application of GA_3_. As shown in Fig. 5, PP_333_ can significantly inhibit the elongation of wild-type catkins, and the effect was enhanced with the increase of PP_333_ concentrations. These results indicated that inhibition of gibberellin biosynthesis could reduce the elongation of wild-type catkins.

### Lower Expression of *KAO* may be Responsible for the Bud Mutation

**Figure 6:**
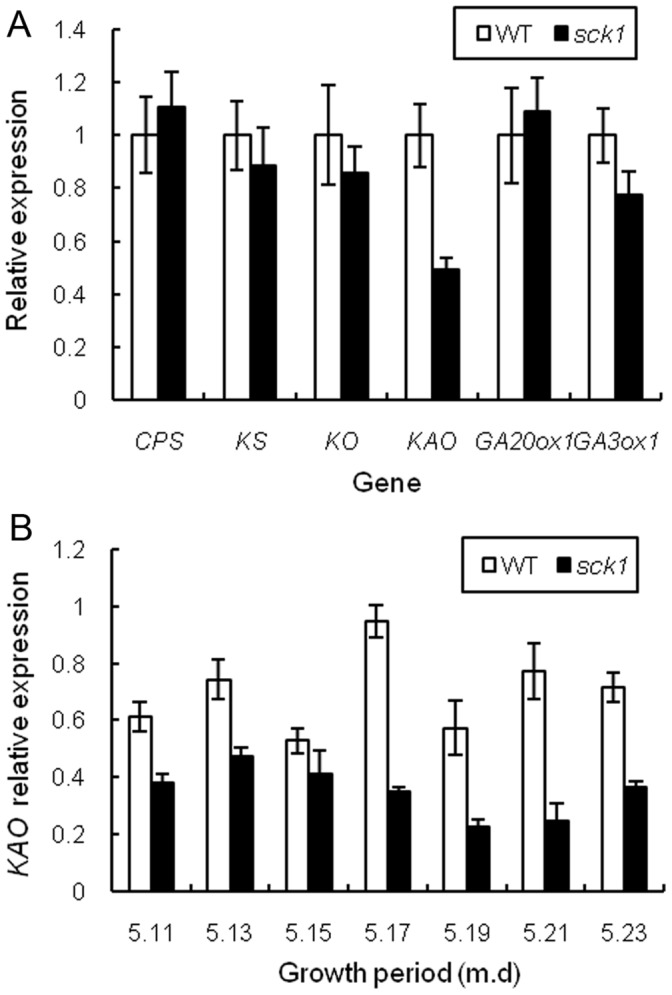
mRNA expression levels of genes involved in gibberellin biosynthetic pathway by real-time PCR. (**A**) mRNA expression levels of *CPS*, *KS*, *KO*, *KAO*, *GA20ox1* and *GA3ox1* involved in WT and *sck1* determined by real-time PCR. The values are expressed relative to the level of transcripts in WT set to be one. (**B**) Comparison of relative expression of *KAO* between WT and *sck1*. Catkins were gathered every two days during growth period in 2010 and the mRNA expression level was determined by RT-PCR. Data are means (±SE, n = 3).

Using quantitative real time PCR (RT-PCR), we further compared the expressions of representative GA biosynthesis-related genes in the primordium formation of the floral cluster, such as *ent*-copalyl diphosphate synthase (*CPS*, HQ658170), *ent*-kaurene synthase (*KS*, HQ658171), *ent*-kaurene oxidase (*KO*, HQ658172), *KAO*, GA 20-oxidase 1 (*GA20ox1*, JN542465) and GA 3-oxidase 1 (*GA3ox1*, JN542466) in *sck1* (Fig. 6 A). Interestingly, the expression of *KAO* was down regulated in *sck1*. In *sck1*, the transcript level of *KAO*, encoding a protein that catalyzes the oxidation of *ent*-kaurenoic acid, was about 0.5 fold of the wild-type by an unknown mechanism. Whereas, the comparative expressions in WT and *sck1* were analyzed and no significant difference of *CPS*, *KS*, *KO*, *GA20ox1* and *GA3ox1* were detected in developing catkins between the WT and *sck1*. In addition, except for May 15 in 2010, the expression of *KAO* in *sck1* was significantly lower (*P*<0.01) than that in WT, which catkins were gathered every two days from May 11 to 23 in 2010 (Fig. 6 B). Those results suggested that the short catkin should be due to the lower expression level of *KAO*.

### Sequence Alignment of the Coding Sequence and Promoter Region of *KAO* in WT and *Sck1*

**Figure 7:**
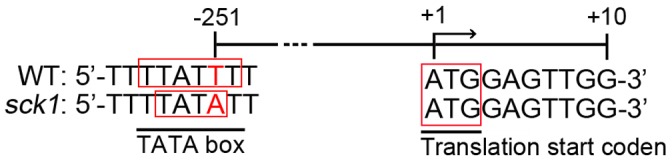
The promoter mutant site of *CmKAO* in *sck1.*

By sequence alignment of the coding region of *KAO* between WT and *sck1*, the result revealed that there was no base mutation. The promoter of *KAO* was cloned by Tail-PCR, and got about 2 000 bp sequence before translation start code. It is found that the T converts to A at the upstream of translation initiation site in the *sck1* (Fig. 7). The TATA box was predicted in http://bioinformatics.psb.ugent.be/webtools/plantcare/html/and http://www.dna.affrc.go.jp/PLACE/, and the sequence of TATA box in WT is TTATTT, but TATA in *sck1*.

### Transient Over-expression of *CmKAO* Partially Rescue the Short Catkins Phenotype in *Sck1*

**Figure 8:**
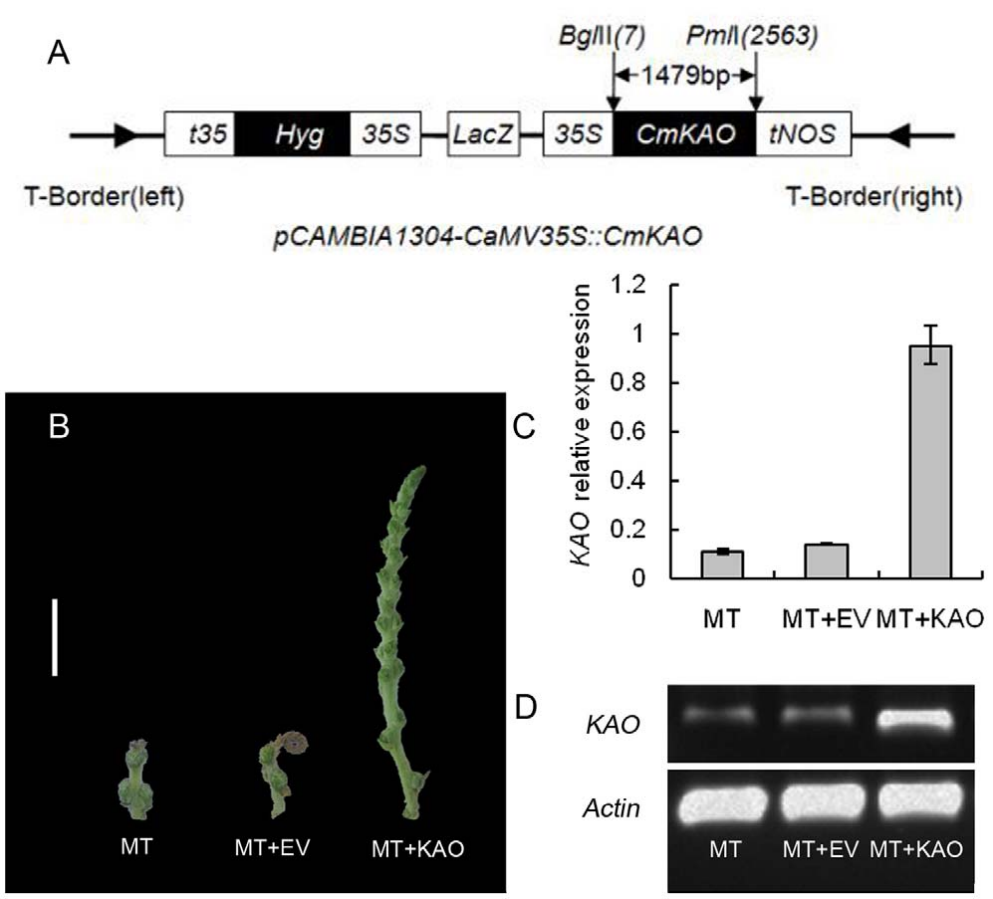
Transient over-expression of *CmKAO* rescued the short catkins phenotype in *sck1*. (**A**) The construct of transforming vector *pCAMBIA1304-CaMV35S::CmKAO*. The *CmKAO* coding sequence (1479 bp) is digested with *Bgl*II and *Pml*I and cloned into the pCAMBIA1304 vector. (**B**) The phenotypes of MT, MT+EV and MT+KAO in the primordium of the stamen, Bar  = 1 cm. (**C**) Comparison of relative expression of *KAO* on MT, MT+EV and MT+KAO. Data are means (±SE, n = 3). (**D**) Semi-quantitative RT-PCR analysis for *CmKAO* transcripts accumulation in MT, MT+EV and MT+KAO. Amplification of *actin* gene transcript was used as an internal control. The RT-PCR products were resolved on cyanine dye-stained 1.0% (w/v) TBE-agarose gels and visualized under UV light.

To further investigate the potential roles of *CmKAO* in *sck1*, we constructed over-expression vector of *CmKAO*, pCAMBIA1304-*CmKAO*. The pCAMBIA1304-*CmKAO* (MT+KAO) and pCAMBIA1304 empty vector (MT+EV) were transformed into *sck1* catkins in the primordium formation of the male inflorescence, and catkins that were transformed with the infiltration buffer were named MT. Catkins of MT and MT+EV began to appear on PCD after about 20 days. We carried out sampling in formation of the primordium of the stamen (Fig. 8 B), when catkins of MT and MT+EV had appeared on programmed cell death and catkins on of MT+KAO did not occur. CaMV35S was verified in MT+EV and MT+KAO; it showed that catkins had been infected by *Agrobacterium*. Real-time PCR analyses were carried out with MT, MT+EV and MT+KAO. The expression of *CmKAO* was similar in MT and MT+EV but was significantly high (*P*<0.01) in MT+KAO (Fig. 8 C). High level of *KAO* transcript was detected in MT+KAO, by contrast, slight level *KAO* transcript signal was detected in MT and MT+EV through semi-quantitative RT-PCR analysis (Fig. 8 D).

### Transient RNA Interference of *CmKAO* in the Wild-type Catkins

**Figure 9:**
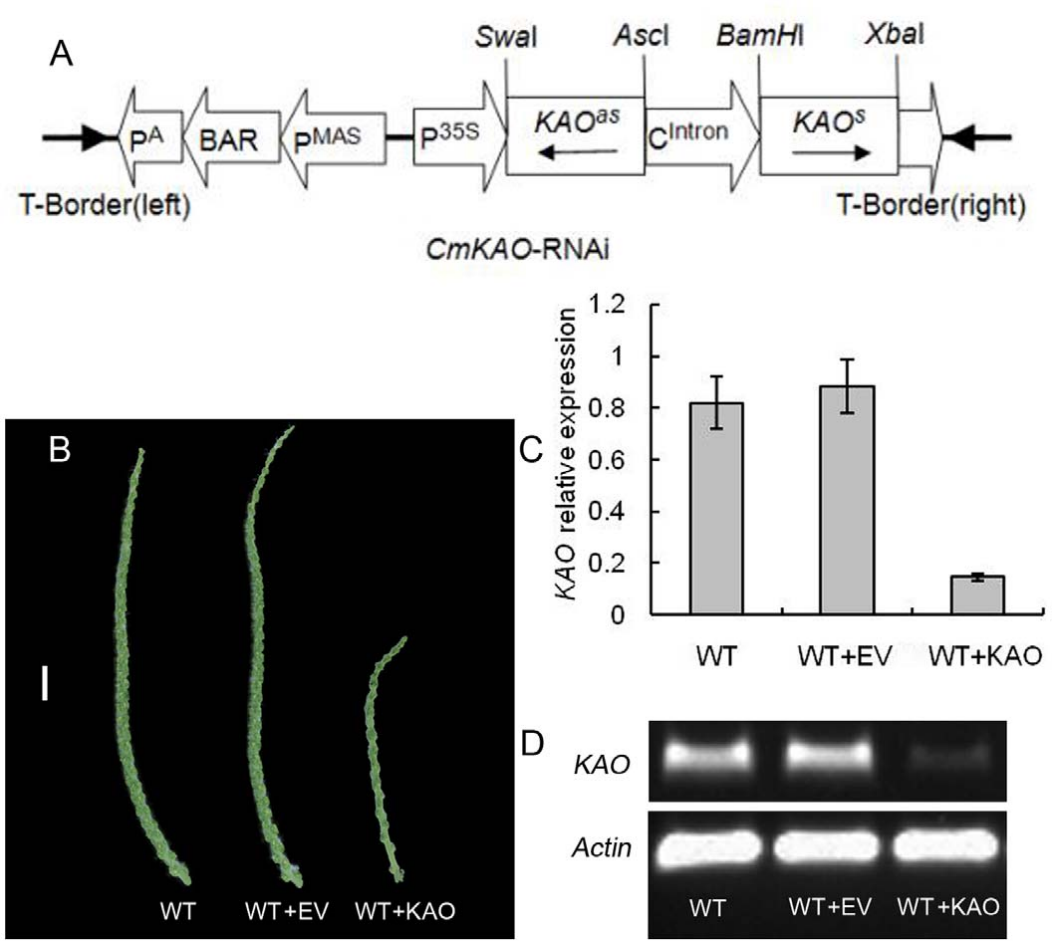
Transient RNA interference of *CmKAO* in the wild-type catkins. (**A**) Production of the *CmKAO*-RNAi cassette. (**B**) The phenotypes of WT, WT+EV and WT+KAO in the primordium of the stamen, Bar  = 1 cm. (**C**) Comparison of relative expression of *KAO* on WT, WT+EV and WT+KAO. Data are means (±SE, n = 3). (**D**) Semi-quantitative RT-PCR analysis for *CmKAO* transcripts accumulation in WT, WT+EV and WT+KAO.

*CmKAO*-RNAi (WT+KAO) vector was constructed based on pFGC5941 and transformed into wild-type catkins in the primordium formation of the male inflorescence. When sampling was carried out in formation of the primordium of the stamen, the catkin length of WT+KAO was about half of two controls’, empty vector WT+EV and WT transformed with infiltration buffer only (Fig. 9 B). For *CmKAO* expression levels, a significantly lower (*P*<0.01) was observed in WT+KAO by Real-time PCR and semi-quantitative RT-PCR, while WT+EV and WT share similar levels (Fig. 9 C, D). Those results showed that the decrease in *KAO* expression has strongly inhibited catkin’s elongation development, and *KAO* was indispensable for catkin development.

## Discussion

Gibberellins (GAs) form a large family of tetracyclic diterpenoid phytohormones that mediate environmental and developmental signals acting by regulating cellular processes such as cell elongation and division. These processes manifest themselves in many aspects of plant growth and development including, seed germination, stem extension, flower initiation and development, and fruit growth [10], [11], [21]–[24]. GA biosynthesis is catalyzed by three classes of enzymes: terpene cyclases catalyze the synthesis of *ent*-kaurene from geranylgeranyl diphosphate; cytochrome P450 monooxygenases catalyze the steps of the pathway from *ent*-kaurene to GA_12_; and soluble dioxygenases catalyze the final steps of the pathway. In the second stage of the pathway, *ent*-kaurene oxidase (KO) catalyze the three-step oxidation of *ent*-kaurene to *ent*-kaurenoic acid (KA) and the enzyme *ent*-kaurenoic acid oxidase (KAO) catalyze the three-step of the GA biosynthesis pathway from KA to GA_12_ in plants.

Dwarf mutants in plants are crucial to elucidate regulatory mechanisms for plant growth and development. This character is also favoured in breeding. The introduction of dwarfing traits into wheat and rice represented a key step to make sure the spectacular increase in food production, obtained since the 1960 s [25], [26]. Identification of the genes responsible for these traits showed that they control gibberellin metabolism and/or perception [25], [27]. Dwarf mutants have been extensively analyzed for their inheritance and their response to plant hormones. There are various reasons for their dwarf phenotypes, associated with, for example, gibberellins [11], [23], brassinosteroids [28], abnormal cell walls [29] and abnormal cell elongation [29], [30]. Dwarf mutants deficient in endogenous GAs have been described for several plant species [11], [24], [26], [31], [32].

Chinese chestnut is one of the important fruit native resources in China but its yield is limited because of lots of male flowers and a few female flowers. One aim in chestnut breeding is to select less male flowers and the high ratio of female to male inflorescences. Short catkins contribute to increase in production of chestnut production and breeding by reducing the nutrient consumption of trees. We have reported that the *sck1* is a short catkins mutation which is a rare germplasm resource in chestnut [4], [33]. We investigated the hormone levels in the *sck1* and it is shown that lower endogenous GAs levels but not other phytohormones. In maize, tomato, rice, sunflower and *Arabidopsis*, severe reductions in endogenous GA levels or GA response prevent normal anther, pollen development and pollen tube growth [26], [34]–[37]. It is very interested that except for short catkins, there are not any other significant different phenotypes between the *sck1* and wild-type in leaf size, phenophase, morphology of bur, nut rate, weight and quality of nut [33], and the endogenous GAs levels are normal in vegetative part and lower in reproductive organ, but its mechanism is not clear. The endogenous levels of GAs and the response to exogenous hormone treatments suggested that the *sck1* was likely impaired in GA biosynthesis. The exact size of the chestnut genome is unknown, but it’s thought to fall somewhere between the smallest plant genome (that of *Arabidopsis*, which has twenty-seven thousand genes) and one of the larger genomes (such as rice, which has around forty-five thousand genes) [38]. According the information from *Fagaceae* database, we analyzed that there is a small gene family of *KAO* candidates in chestnut and we isolated one from Chinese chestnut. It has high similarity to *Lactuca sativa LsKAO1* [39], *AtKAO1* and *AtKAO2* of *Arabidopsis* [40], *CmKAO1* of pumpkin [41] and the two *KAO* genes of pea [42].

There is no difference in gene coding region of *KAO* between wild-type and the *sck1*. It is very interested that one point mutation was found in the promoter region of *KAO*, and the T convert to A at the upstream of translation initiation site in the *sck1*. We analysed the mutation region by bioinformatics method and predicted that the point mutation may be a TATA box in the upstream of translation initiation site. The mutation may cause the decrease of the transcript of *KAO* in the *sck1*. But because of the requirement of a long incubation time from transformant screening to bearing fruit, it will spend us long time to confirm the function of the promoter of *KAO*.

It is very difficult to establish a stable transgenic expression system in fruit trees. However, this is the key constraint to the development of molecular biology in fruit trees. The transgenic technology through embryogenesis on American chestnut and European chestnut had established [43], [44]. Embryogenesis of *C. mollissima* is few reported [45], because the most tissue culture of Chinese chestnut is organogenesis and the transgenic on Chinese chestnut had not been reported. We try to establish a kind of transgenic transient expression method in chestnut. The transient over-expressing *KAO* in the *sck1* can partially rescue the short catkins by average 4–5 fold and could avoid the programmed cell death. Although we don’t know the mechanism, it may be caused by the increase the *KAO* expression in the early transient expression. In addition, the transient RNA interference of *CmKAO* in the wild-type catkin can inhibit the wild-type catkins elongation strongly.

## Materials and Methods

### Field Studies

All necessary permits were obtained for the described field studies. In addition, the field studies did not involve endangered or protected species.

### Plant Materials

The *Castanea mollissima* mutant, *sck1* in a mountainous area near Beijing, China, has been registered in State Forestry Administration, P. R. China (Varieties No. 20090015) [46]. The materials for determination of GAs contents and relative expressions of representative GA biosynthesis-related genes were collected from both *sck1* and WT. Phytohormone feeding and transgenic experiment was conducted in the trees which were grafted with the mutant catkins phenotype in 2000, and PP_333_ treatment was conducted in 7-year-old wild-type chestnut. All of grafted trees were grown in the same orchard, and subjected to standard cultivation. The catkins were separated from sampled trees, immediately frozen in liquid nitrogen, and kept at −80°C until analysed.

### Determination of Endogenous GAs Content

Fresh catkins (0.5 g) were immediately weighed, frozen in liquid nitrogen and later lyophilized. Each replicate sample was extracted in 80% MeOH with internal standards of [^2^H_2_]-GA_1_, [^2^H_2_]-GA_3_, [^2^H_2_]-GA_4_, [^2^H_2_]-GA_7_, [^2^H_2_]-GA_12_ [^2^H_2_]-GA_24_, [^2^H_2_]-GA_44_ overnight and reduced to aqueous phase by vacuum. The aqueous phase was adjust to pH 3.0 and partitioned with EtOAc (Merck, Darmstadt, Germany), and then the organic phase was repartitioned with K-Pi buffer (pH 8.5) (Merck), and the aqueous phase was adjusted to pH 3.0 and partitioned again with EtOAc. Further purification was performed on QAE Sephadex A-25 and C_18_ Sep-Pak. The eluate was dried and redissolved with high-performance liquid chromatography (HPLC) initial solution and filtered through a 0.22 µm filter and analyzed with an LC-MS system (LCQ Deca MAX, HPLC-ESI-MS, Thermo-Finnigan, Ringoes, NJ, USA). MS–MS data were then analyzed using the software Xcalibur 2.1 (Thermo-Finnigan) and quantified by reference to ratios of specific ions of phytohormones in natural samples over those of internal standards using equations for isotope dilution analysis.

### Feeding Experiment

GA_3_ was diluted with distill water to 150, 250, 350 mg/L. At early developmental stage of catkins, the short catkins on 7-year-old trees selected from the top of the crown periphery in the same direction of the mutant branch were sprayed with different concentrations of GA_3_ every two days. Distilled water was used as control, and selected for each treatment of 30 catkins.

PP_333_ was diluted with distill water to 500, 1000, 1500 mg/L, the selection of wild-type catkins on 7-year-old trees and treatment were the same as described in GA_3_ treatment.

### RNA Extraction, cDNA Synthesis and Cloning of *CPS*, *KS*, *KO*, *KAO*, *GA20ox1* and *GA3ox1*

Total RNA was isolated from catkins using EASYspin plant RNA extract Kit (Biomed, Beijing, China). All RNA were treated and purified with DNAase I (Tiangen, Beijing, China) to remove genomic DNA contamination. First-strand cDNA was synthesized from total RNA using Reverse Transcriptase M-MLV (H^−^) (TaKaRa Bio, Ohtsu, Japan). The reverse transcription reaction mixture contained 1 µg of total RNA, 1 µL Oligo d(T)_18_ primer (10 µmol/L) and diethylpyrocarbonate (DEPC) water up to 6 µL. The mixture was incubated at 72°C for 10 min and 0°C for 3 min, followed by the addition of 2 µL 5×reverse transcriptase buffer, 2 µL 2.5 mmol/L of each dNTP, 0.25 µL Ribonuclease Inhibitor (RRI) and 5 U Reverse Transcriptase M–MLV (H^−^). The mixture was incubated at 42°C for 1 h and inactivated at 72°C for 15 min.

According to the conserved nucleotide sequences in other plants, the degenerative primers of *CPS*, *KS*, *KO*, *KAO*, *GA20ox1* and *GA3ox1* were designed, and DNA fragments were amplified from templates of cDNA and sequenced. Full-length cDNA was obtained for *CPS*, *KS*, *KO*, *KAO*, *GA20ox1* and *GA3ox1* by RACE using SMART™ cDNA Library Construction Kit (TaKaRa Bio) with nested PCR according to the manufacturer’s instructions. For 3′ RACE and 5′ RACE, the gene-specific primers in the first and second reactions were designed based on above partial sequence. A full-length cDNA clone was obtained by PCR according to the manufacturer’s instructions using gene-specific primers. All above products were isolated by electrophoresis through agarose and cloned in pMD19-T vector, and then sequenced (Sangon, Shanghai, China).

### Real-time PCR

Primers were designed with the Primer 5.0 software and following the manufacturer’s guidelines for primer design. All primers used to real-time PCR were showed in table 1. *Actin* was amplified along with the target gene as an endogenous control to normalize expression among different samples. Real-time PCR was performed using the Bio-RAD C1000™ Thermal Cycler with CFX96™ Real Time System. The reaction mix was prepared following the SYBR Premix Ex Taq™ manufacturer (TaKaRa Bio). The amplification condition was as follows: 95°C for 2 min; 40 cycles of 94°C for 20 s, 54°C for 20 s, and 72°C for 20 s. PCR data were collected and analyzed with the CFX manager software version 1.5 and the 2^−ΔΔCt^ method [47], and each sample was detected for 3 duplicates. The relative expression levels of the genes of interest were calculated relative to the expression of *actin*. Microsoft Excel program was employed to depict expression data.

**Table 1:** Primers designed using Primer 5.0 software and used to real-time PCR analysis of *CPS*, *KS*, *KO*, *KAO*, *GA20ox1*and *GA3ox1* expression.

Gene	Sense primer	Antisense primer
*Actin*	5′-TTGACTATGAGCAGGAACTT-3′	5′-TTGTAGGTGGTCTCGTGAAT-3′
*CPS*	5′-GGTGTTAGGCAGCAAGGAG-3′	5′-TAATGAGGTCAGATAGTGGC-3′
*KS*	5′-GGCTACAAGGTGAGGAGGAA-3′	5′-CCAAGGGAACTGGAGAAATG-3′
*KO*	5′-AAAGGGTGCGATTGAGGT-3′	5′-CAGTAGTATCCGCCGTCT-3′
*KAO*	5′-CTTTCTAAGGTGATTGACGA-3′	5′-GCTCCTGCTTTGGGTGTA-3′
*GA20ox1*	5′-GGTTTACCAGGACTATTGCG-3′	5′-ATGAAGGTGTCTCCGATGTT-3′
*GA3ox1*	5′-TTATGTGGCTAATCTTGGGCTC-3′	5′-CTGGTGTTGTTTTGGTGGAGAAT-3′

### Obtaining Sequence of Coding and Promoter Region of *CmKAO*

The coding sequence of *CmKAO* was obtained by PCR from wild-type and mutant cDNA using a LA Taq and the *CmKAO*-specific primers KAOs (forward, 5′-GGAAGATCTGATGGAGTTGG GTCCTATC-3′) and KAOa (reverse, 5′-GGA TCCACGTGTTAGAGAGAAGAAGAGGG-3′).

The thermal asymetric interlaced (Tail)-PCR protocol was modified for high throughput as follows. A *KAO* border-specific primer and a pool of two arbitrary degenerate (AD) primers (AD1, 5′- WGTGNAGWANCANAGA -3′; AD2, 5′- TGWGNAGSANCASAGA -3′ were used per round of Tail-PCR cycling. The *KAO* border primers used were as follows: LB1 (5′- ATGTTTGC CTGAATAACAATAAACTAGAACATGAC -3′), LB2 (5′-AAACTAGAACATGACTTAATA TTCAGAGGAGAG -3′), or LB3 (5′- AAACAAAGGAGGAGACGAAAGAATCAGG -3′); Third of rounds of Tail-PCR cycling were performed.

Cycling parameters for the first round were as follows: (1) 94°C for 3 min and 95°C for 2 min; (2) 5 Cycles of 94°C for 1 min, 62°C for 1 min, and 72°C for 2.5 min; (3) 94°C for 30 s, 25°C for 30 s, 30°C for 30 s, 35°C for 30 s, 40°C for 30 s, 45°C for 30 s, 50°C for 30 s, 55°C for 30 s, 60°C for 30 s, 65°C for 30 s, and 72°C for 2.5 min; (4) 15 cycles of 94°C for 30 s, 62°C for 1 min, 72°C for 2.5 min, 94°C for 30 s, 62°C for 1 min, 72°C for 2.5 min, 94°C for 30 s, 44°C for 1 min, and 72°C for 2.5 min; and (5) 72°C for 10 min. Cycling parameters for the second round were as follows: (1) 94°C for 3 min and 95°C for 2 min; (2) 15 cycles of 94°C for 30 s, 62°C for 1 min, 72°C for 2.5 min, 94°C for 30 s, 62°C for 1 min, 72°C for 2.5 min, 94°C for 10 s, 44°C for 1 min, and 72°C for 2.5 min; (3) 72°C for 10 min. Cycling parameters for the third round were as follows: (1) 94°C for 3 min; (2) 15 cycles of 94°C for 30 s, 62°C for 1 min, 72°C for 2.5 min, 94°C for 30 s, 62°C for 1 min, 72°C for 2.5 min, 94°C for 10 s, 44°C for 1 min, and 72°C for 2.5 min; (3) 72°C for 10 min. LA Taq polymerase (TaKaRa Bio, Ohtsu, Japan) was used for all amplifications. The primary Tail reaction contained 5 ng of genomic DNA.

### *Agrobacterium*-mediated Transformation of the Short Catkins with Wild-type *CmKAO* CDNA

Whole coding sequence of *CmKAO* was obtained by PCR from wild-type cDNA using a LA Taq and the *CmKAO*-specific primers KAOs with a *Bgl*II restriction site and KAOa with a *Pml*I restriction site. The sequence was cloned into pMD-19T Vector to generate the entry vector pMD-19T-CmKAO. This entry vector was recombined into the destination vector pCAMBIA1304 (pre-digested by *Bgl*II and *Pml*I) downstream of the 35S promoter to generate the recombinant plasmid *pCAMBIA1304-CaMV35S::CmKAO* (Fig. 8 A). The construct was transferred into *Agrobacterium tumefaciens* GV3101, using the freeze-thaw method.

Single colonies of *Agrobacterium* harboring the pCAMBIA 1304-CmKAO vector were grown overnight at 28°C with vigorous shaking (180–200 rpm) in 1 mL of LB liquid medium supplemented with 50 mg L^−1^ rifampicin, 50 mg L^−1^ kanamycin. 1 mL of the culture was used to inoculate, in 250 Erlenmeyer flasks, 100 mL of LB medium supplemented with the appropriate antibiotics plus 10 mM 2−(4-Morpholino) ethanesulfonic acid and 20 µM acetosyringone. The cultures were incubated for 12 h at 28°C with vigorous shaking. When the culture reached an OD600 of about 0.8, *Agrobacterium* were collected by centrifuging the culture in polypropylene tubes at 2,000 g for 10 min at room temperature. The cells were gently resuspended in 10 mL of infiltration buffer (LB medium supplemented with 10 mM MgCl_2_, 10 mM 2−(4-Morpholino) ethanesulfonic acid and 200 µM acetosyringone), once resuspended, the cultures were used to prepare 30 mL of bacterial suspension in LB medium a 1.5 OD (600 nm). The cultures were incubated for 3 h at room temperature with gentle shaking. The cultures were evenly injected throughout the short catkins by using a sterile 1 mL hypodermic syringe and the short catkins were soaked with writing brush soon (MT+KAO). the short catkins were transformed with an *Agrobacterium* harboring the pCAMBIA 1304 vector without the whole coding sequence of *CmKAO* cDNA and using the same procedure above described (MT+EV). Agroinoculation and syringe inoculation were based on previous method [48].

### Semi-quantitative RT-PCR Analysis

To analyse MT, MT+EV and MT+KAO transcript levels, First strand cDNAs derived from 1 µg of total RNA (see “RNA extraction") were used in all PCR amplifications. PCRs were performed using gene-specific primers for *CmKAO*. To normalize the amount of RNA in each sample, an amplification of the constitutively expressed chestnut *actin* transcript was carried out. The number of PCR cycles was chosen in the exponential range of amplification. Primers (Table 1) were designed to yield the 217- and 178-bp fragments for *CmKAO* and *actin*, respectively. The PCR conditions were: 95°C for 3 min, 28cycles (30 s at 94°C, 30 s at 54°C, 20 s at 72°C), with a final extension of 5 min at 72°C. All PCR products were separated by electrophoresis on a 1% TBE-agarose gel and visualized with cyanine dye under UV light.

### Transient RNA Interference of *CmKAO* in the Wild-type Catkins

Primers used for amplification of *CmKAO* were KAOsf with *BamH*I (5′-GGATCCTGAAATG GGTTGTGAAGAATG-3′) and KAOsr with *Xba*I (5′-TCTAGACGTCTATCAAAGCGTCC ATC-3′) for the sense orientation and KAOaf with *Swa*I (5′-ATTTAAATTGAAATGGGTTG TGAAGAATG-3′) and KAOar with *Asc*I (5′-GGCGCGCCCGTCTATCAAAGCGTCC ATC-3′) for the anti-sense orientation. Purified amplification products were ligated into PMD-19-T Vector (TaKaRa) and the complete sequence of the ‘‘sense’’ clone was determined. Subsequently the separate sense and anti-sense fragments were transferred into the RNAi vector pFGC5941, as described in Figure 9. The construct was transferred into *Agrobacterium tumefaciens* EHA105, using the freeze-thaw method. The wild-type catkins injected and soaked through *Agrobacterium*-mediated transformation.

### Statistical Analysis

The data were subjected to statistic analysis, and analysis of variance was performed using the SPSS 13.0 program.

## References

[1] Feng ZQ, Jiao ZG, Zhang DS (1995) Study of reason on thinning catkins for Chinese chestnut. Chinese Fruit 1: 14–15.

[2] Liu K, Zhao Z, Li C (1999) Effect of thinning catkins on nutrition in the Chinese chestnut tree. ISHS Acta Hort 494: 191–194.

[3] Zhao ZY, Liu KY (2009) Effect of chemical thinning catkins on Chinese chestnut yield and quality. ISHS Acta Hort 844: 457–460.

[4] Feng YQ, Shen YY, Qin L, Cao QQ, Han ZH (2011) Short catkin1, a novel mutant of Castanea mollissima, is associated with programmed cell death during chestnut staminate flower differentiation. Sci Hortic-Amsterdam 130: 434–435.

[5] Yao JL, Dong YH, Morris BAM (2001) Parthenocarpic apple fruit production conferred by transposon insertion muations in a MADS-box transcription factor. P Natl Acad Sci USA 98: 1306–1311. 10.1073/pnas.031502498 PMC14750 11158635

[6] Kobayashi S, Yamamoto NG, Hirochika H (2004) Retrotransposon-induced mutations in grape skin color. Science 304: 982. 15143274 10.1126/science.1095011

[7] De Schepper S, Van Bockstaele E, Debergh P, De Loose M (2001a) Molecular analysis of sports induction in azalea (Rhododendron simsii hybrids). Acta Hort 552: 143–149.

[8] Han XY, Wang LS, Liu ZA, Jan DR, Shu QY (2008) Characterization of sequence-related amplified polymorphism markers analysis of tree peony bud sports. Sci Hortic-Amsterdam 115: 261–267.

[9] Hedden P, Phillips AL (2000) Gibberellin metabolism: new insights revealed by the genes. Trends Plant Sci 5: 523–530. 11120474 10.1016/s1360-1385(00)01790-8

[10] Olszewski N, Sun TP, Gubler F (2002) Gibberellin signaling: biosynthesis, catabolism, and response pathways. Plant Cell (Supplement): S61–S80. 10.1105/tpc.010476 PMC151248 12045270

[11] Yamaguchi S (2008) Gibberellin metabolism and its regulation. Annu Rev Plant Biol 59: 225–251. 18173378 10.1146/annurev.arplant.59.032607.092804

[12] Koornneef M, van der Veen JH (1980) Induction and analysis of gibberellin sensitive mutants in Arabidopsis thaliana (L.) Heynh. Theor Appl genet 58: 257–263. 24301503 10.1007/BF00265176

[13] Sun TP, Kamiya Y (1994) The arabidopsis GA1 locus encodes the cyclase ent-Kaurene synthetase A of gibberellin biosynthesis. Plant Cell 6: 1509–1518. 7994182 10.1105/tpc.6.10.1509 PMC160538

[14] Chiang HH, Hwang I, Goodman HM (1995) Isolation of the arabidopsis GA_4_ locus. Plant Cell 7: 195. 7756830 10.1105/tpc.7.2.195 PMC160775

[15] Phillips AL, Ward DA, Uknes S, Appleford NEJ, Lange T, et al (1995) Isolation and expression of three gibberellin 20-Oxidase cDNA clones from arabidopsis. Plant Physiol 108: 1049–1057. 7630935 10.1104/pp.108.3.1049 PMC157456

[16] Yamaguchi S, Sun TP, Kawaide H, Kamiya Y (1998) The GA2 locus of arabidopsis thaliana encodes ent-Kaurene synthase of gibberellin biosynthesis. Plant Physiol 116: 1271–1278. 9536043 10.1104/pp.116.4.1271 PMC35033

[17] Davidson SE, Reid JB, Helliwell CA (2006) Cytochromes P450 in gibberellin biosynthesis. Phytochem Rev 5: 405–419.

[18] Boss PK, Thomas MR (2002) Association of dwarfism and floral induction with a grape “green revolution" mutation. Nature 416: 847–849. 11976683 10.1038/416847a

[19] Li XL, Guo XP, Shen YY, Cao QQ, Feng YQ, et al (2011) Preliminary identification of GAs-deficient short male catkin mutant and expression analysis of CmGID1 in Castanea mollissima. Acta Horticulturae Sinica 38(7): 1251–1258.

[20] Hedden P, Graebe JE (1985) Inhibition of gibberellin biosynthesis by paclobutrazol in cell-free homogenates of Cucurbita maxima endosperm and Malus pumila embryos. J Plant Growth Regul 4: 1–4.

[21] Fleet CM, Sun TP (2005) A DELLAcate balance: the role of gibberellin in plant morphogenesis. Curr Opin Plant Biol 8: 77–85. 15653404 10.1016/j.pbi.2004.11.015

[22] Hedden P (1999) Recent advances in gibberellin biosynthesis. J Exp Bot 50: 553–563.

[23] Swain SM, Singh DP (2005) Tall tales from sly dwarves: novel functions of gibberellins in plant development. Trends Plant Sci 10: 123–129. 15749470 10.1016/j.tplants.2005.01.007

[24] Yamaguchi S (2006) Gibberellin biosynthesis in Arabidopsis. Phytochem Rev 5: 39–47.

[25] Hedden P (2003) The genes of the green revolution. Trends Genet 19: 5–9. 12493241 10.1016/s0168-9525(02)00009-4

[26] Sakamoto T, Matsuoka M (2004) Generating high-yielding varieties by genetic manipulation of plant architecture. Curr Opin Biotech 15: 144–147. 15081053 10.1016/j.copbio.2004.02.003

[27] Peng JR, Richards DE, Hartley NM, Murphy GP, Devos KM (1999) ‘‘Green revolution’’ genes encode mutant gibberellin response modulators. Nature 400: 256–261. 10421366 10.1038/22307

[28] Bishop GJ, Koncz C (2002) Brassinosteroids and plant steroid hormone signalling. Plant Cell 14: S97–S110. 12045272 10.1105/tpc.001461 PMC151250

[29] Darley CP, Forrester AM, MacQueen-Mason SJ (2001) The molecular basis of plant cell wall extension. Plant Mol Biol 47: 179–195. 11554471

[30] Buluška F, Busti E, Dolfini S, Gavazzi G, Volkmann D (2001) Lilliputian mutant of maize lacks cell elongation and shows defects in organization of actin cytoskeleton. Dev Biol 236: 478–491. 11476586 10.1006/dbio.2001.0333

[31] Koornneef M, Bosma TDG, Hanhart CJ, van der Veen JH, Zeevaart JAD (1990) The isolation and characterization of gibberellin-deficient mutants in tomato. Theor Appl Genet 80: 852–857. 24221121 10.1007/BF00224204

[32] Ross JJ, Murfet IC, Reid JB (1997) Gibberellin mutants. Physiol Plantarum 100: 550–560.

[33] Feng YQ, Qin L, Yang DS, Dong QH (2005) Study on the main characteristics of shorter male inflorescence sport of Chinese chestnut. Journal of Beijing Agricultural College 20: 1–5.

[34] Cecconi F, Gaetani M, Lenzi C, Durante M (2002) The sunflower dwarf mutant dw1: effects of gibberellic acid treatments. Helia 36: 161–166.

[35] Chhun T, Aya K, Asano K, Yamamoto E, Morinaka Y (2007) Gibberellin regulates pollen viability and pollen tube growth in rice. Plant Cell 19: 3876–3888. 18083909 10.1105/tpc.107.054759 PMC2217639

[36] Margis-Pinheiro M, Zhou XR, Zhu QH, Dennis ES, Upadhyaya NM (2005) Isolation and characterization of a Ds-tagged rice (Oryza sativa L.) GA-responsive dwarf mutant defective in an early step of the gibberellin biosynthesis pathway. Plant Cell Rep 23: 819–833. 15668792 10.1007/s00299-004-0896-6

[37] Singh DP, Jermakow AM, Swain SM (2002) Gibberellins are required for seed development and pollen tube growth in Arabidopsis. Plant Cell 14: 3133–3147. 12468732 10.1105/tpc.003046 PMC151207

[38] Freinkel S (2009) American chestnut: The life, death, and rebirth of a perfect tree. Berkeley, CA: University of California Press.

[39] Sawada Y, Katsumata T, Kitamura J, Kawaide H, Nakajima M (2008) Germination of photoblastic lettuce seeds is regulated via the control of endogenous physiologically active gibberellin content, rather than of gibberellin responsiveness. J Exp Bot 59: 3383–3393. 18653696 10.1093/jxb/ern192 PMC2529229

[40] Helliwell CA, Chandler PM, Poole A, Dennis ES, Peacock WJ (2001) The CYP88A cytochrome P450, ent-kaurenoic acid oxidase, catalyzes three steps of the gibberellin biosynthesis pathway. P Natl Acad Sci USA 98: 2065–2070. 10.1073/pnas.041588998 PMC29382 11172076

[41] Helliwell CA, Olive MR, Gebbie L, Forster R, Peacock WJ (2000) Isolation of an ent-kaurene oxidase cDNA from Cucurbita maxima. Aust J Plant Physiol 27: 1141–1149.

[42] Davidson SE, Elliott RC, Helliwell CA, Poole AT, Reid JB (2003) The pea gene NA encodes ent-kaurenoic acid oxidase. Plant Physiol. 131: 335–344. 10.1104/pp.012963 PMC166813 12529541

[43] Andrade GM, Nairn CJ, Le HT, Merkle SA (2009) Sexually mature transgenic American chestnut trees via embryogenic suspension-based transformation. Plant Cell Rep 28: 1385–1397. 19578855 10.1007/s00299-009-0738-7

[44] Seabra RC, Pais MS (1998) Genetic transformation of European chestnut. Plant Cell Rep 17: 177–182. 10.1007/s002990050374 30736496

[45] Zhang L, Yin WL, Wang HF (2007) Ovule culture in vitro of Castanea mollissima Bl. Journal of Beijing Forestry University 29: 99–105.

[46] Qin L, Yang DS, Gao TF, Zhou ZJ, Wang TM, et al. (2011) A new variety of Chinese chestnut ‘Duanhuayunfeng’. Scientia Silvae Sinicae 47(4): 194, 197.

[47] Livak KJ, Schmittgen TD (2001) Analysis of relative gene expression data using real-time quantitative PCR and the 2^−△△Ct^ method. Methods 25: 402–408. 11846609 10.1006/meth.2001.1262

[48] Fu DQ, Zhu BZ, Zhu HL, Jiang WB, Luo YB (2005) Virus-induced gene silencing in tomato fruit. Plant J 43: 299–308. 15998315 10.1111/j.1365-313X.2005.02441.x

